# Intra-Species Genetic Diversity and Clonal Structure of *Cryptosporidium parvum* in Sheep Farms in a Confined Geographical Area in Northeastern Spain

**DOI:** 10.1371/journal.pone.0155336

**Published:** 2016-05-13

**Authors:** Ana Ramo, Luis V. Monteagudo, Emilio Del Cacho, Caridad Sánchez-Acedo, Joaquín Quílez

**Affiliations:** 1 Department of Animal Pathology, Faculty of Veterinary Sciences, University of Zaragoza, Zaragoza, Spain; 2 Department of Anatomy, Embryology and Genetics, Faculty of Veterinary Sciences, University of Zaragoza, Zaragoza, Spain; University of Minnesota, UNITED STATES

## Abstract

A multilocus fragment typing approach including eleven variable-number tandem-repeat (VNTR) loci and the GP60 gene was used to investigate the intra-farm and intra-host genetic diversity of *Cryptosporidium parvum* in sheep farms in a confined area in northeastern Spain. Genomic DNA samples of 113 *C*. *parvum* isolates from diarrheic pre-weaned lambs collected in 49 meat-type sheep farms were analyzed. Loci exhibited various degrees of polymorphism, the finding of 7–9 alleles in the four most variable and discriminatory markers (ML2, Cgd6_5400, Cgd6_3940, and GP60) being remarkable. The combination of alleles at the twelve loci identified a total of 74 multilocus subtypes (MLTs) and provided a Hunter-Gaston discriminatory index of 0.988 (95% CI, 0.979−0.996). The finding that most MLTs (n = 64) were unique to individual farms evidenced that cryptosporidial infection is mainly transmitted within sheep flocks, with herd-to-herd transmission playing a secondary role. Limited intra- host variability was found, since only five isolates were genotypically mixed. In contrast, a significant intra-farm genetic diversity was seen, with the presence of multiple MLTs on more than a half of the farms (28/46), suggesting frequent mutations or genetic exchange through recombination. Comparison with a previous study in calves in northern Spain using the same 12-loci typing approach showed differences in the identity of major alleles at most loci, with a single MLT being shared between lambs and calves. Analysis of evolutionary descent by the algorithm eBURST indicated a high degree of genetic divergence, with over 41% MLTs appearing as singletons along with a high number of clonal complexes, most of them linking only two MLTs. Bayesian Structure analysis and *F* statistics also revealed the genetic remoteness of most *C*. *parvum* isolates and no ancestral population size was chosen. Linkage analysis evidenced a prevalent pattern of clonality within the parasite population.

## Introduction

*Cryptosporidium parvum* is an ubiquitous and significant entero-pathogen causing diarrheal illness in many species of mammals, particularly humans and young livestock. Molecular studies have revealed a complex epidemiological picture for this protozoan, which exhibits an extensive intra-species diversity and potential genetic recombination during the sexual phase of its life cycle [1]. Human-specific, animal-specific and zoonotic subtypes have been identified using sequence analysis of the 60 kDa glycoprotein (GP60) gene, which is the single most polymorphic marker identified so far in the *Cryptosporidium* genome. Nevertheless, the GP60 gene is under selective pressure as it mediates parasite attachment to host cells, and its use as a single locus subtyping method has been reported to underestimate genetic diversity where sexual reproduction occurs [1]. The high variability of short variable-number tandem-repeat (VNTR) loci, also known as minisatellites and microsatellites, has made them the genetic markers of choice for addressing the population structure and transmission dynamics of *C*. *parvum*, being used in either multilocus sequence typing (MLST) or multilocus fragment typing (MLFT) analysis [2].

Most contributions to the molecular epidemiology of *C*. *parvum* in animal hosts have focused on cattle, which is considered the primary non-human species impacted by cryptosporidiosis and the major zoonotic reservoir for humans [2]. In contrast, the genetic variability of this protozoan in sheep remains largely unexplored, although it is the most populous livestock in many countries. *C*. *parvum* is the most prevalent species in diarrheic pre-weaned lambs in most European countries [3–6], but modest numbers of ovine specimens have been genetically characterized using GP60 sequencing [6–11], and particularly other VNTR loci [7, 12–17]. All ovine isolates have been found to belong to the potentially zoonotic subtype families IIa and IId, and both sporadic and outbreak-related human cases involving direct contact with lambs have been documented [18–19].

Sheep production is of great economic significance in Spain, which is the second country with the largest sheep population in the European Union. A total of 15.4 million head of sheep were estimated in 2014 as compared to 6.1 million head of cattle (http://ec.europa.eu/eurostat). Most traditional farms apply a semi-extensive farming system, where adult animals are fed on pasture during the day-time but young animals are kept indoors until weaning. This husbandry system provides good opportunities for the transmission of *Cryptosporidium* and other enteric pathogens either by direct contact or transmission from a heavy contaminated lambing area. In fact, neonatal lamb diarrhea is a prominent and economically devastating condition in sheep farms in Spain, and *Cryptosporidium* spp. is among the most prevalent enteric pathogens associated with this syndrome [20]. *C*. *parvum* is responsible for most of these diarrheic outbreaks, but geographical differences in the distribution of GP60 subtypes have been reported, with the predominance of family IIa in the northwest as compared to family IId in the northeast of the country [21–24].

A previous study in sheep farms in northeastern Spain showed the high discriminatory power of a multilocus approach to differentiate *C*. *parvum* at a local geographical level. The technique combined six VNTR loci and detected a high number of allelic variants, revealing the presence of host-associated subpopulations [25]. The current study aimed to investigate if this genetic variability also applies to much more confined areas. For this purpose, *C*. *parvum* isolates from farms in a single province were analyzed using a MLFT approach. This province was selected based on its high sheep density and endemicity of cryptosporidiosis, with infection rates over 76% in diarrheic lambs aged 8–14 days [26]. Moreover, an expanded panel of markers was used, including eleven VNTR loci and the GP60 subtype, in order to explore in more detail the intra-farm and intra-host genetic diversity and elucidate the population genetic structure of *C*. *parvum* in this population.

## Materials and Methods

### Ethics Statement

*Cryptosporidium* isolates were obtained from feces collected for diagnostic purposes from lambs after the permission of farm owners, with no specific permits being required by the authority for the feces collections. Animal care and use committee approval was not necessary for this study. Directive 2010/63/EU of the European Parliament on the protection of animals used for scientific purposes does not apply to non-experimental clinical veterinary practices.

### Parasite isolates

**Fig 1:**
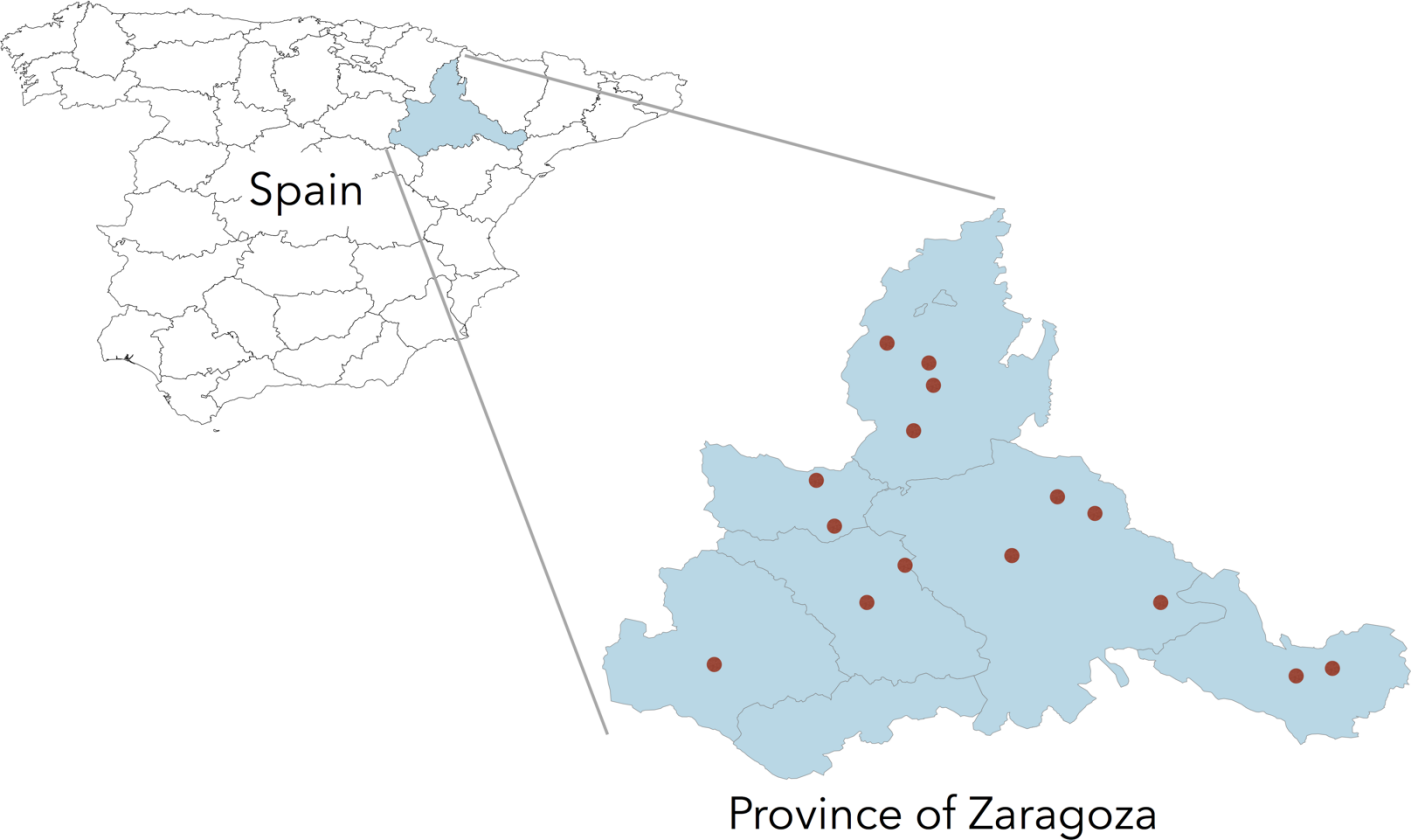
Geographic map of the sampling locations in the province of Zaragoza (NE Spain).

Genomic DNA samples of *Cryptosporidium parvum* isolates from a previous study for the identification of *Cryptosporidium* species and GP60 subtypes infecting domestic ruminants were used [27]. These isolates were obtained between 2009 and 2012 from 113 diarrheic pre-weaned lambs in 49 meat-type sheep farms under semi-extensive management system in the province of Zaragoza, a geographical area which covers approximately 17,274 km^2^ in the northeast of Spain (Fig 1). Production of sheep meat is a major sector in this province, with a total sheep population in 2013 of 540,446 animal and 1,068 farms (Government of Aragon, http://www.aragon.es/). Most flocks in this area breed their own replacement females rather than buying ewe lambs. Most farms were sampled on a single diarrheic outbreak and only one specimen from each lamb was analyzed. *Cryptosporidium* species and *C*. *parvum* GP60 alleles in the previous study were determined based on a fragment size typing approach at the small-subunit (SSU) rRNA and GP60 genes, respectively.

### Automated fragment analysis

Each isolate was subtyped at eleven VNTR markers, including six minisatellites (MSB, MSC6-7, cgd2_3850, cgd1_3670, cgd6_5400 and cgd6_3940) and five microsatellites (ML1, ML2, TP14, 5B12, CP47), using previously described primers and conditions [7, 12, 13, 28–32]. Fluorescently labeled primers were used in order to allocate alleles with overlapping peaks to a specific locus. According to the amplicon intensity, 0.5-to-2 μl samples of the mini- and microsatellite-labeled PCR products for each *C*. *parvum* isolate were then mixed together with 0.3 μl of the standard ladder (GeneScan 600 Liz Size Standard; Applied Biosystems, Life Technologies) and 8.5 μl of deionized formamide. The mixture was then denatured (95°C for 2 min) and subjected to capillary electrophoresis (CE) on a 3500xL Genetic Analyzer (Applied Biosystems, Life Technologies). Data were stored and analyzed with the aid of Gene Mapper software (version 4.1) to determinate fragment sizes. Allele nomenclature was based on the fragment size (in base pairs) adjusted after comparison with reference sequenced material. For this purpose, representative isolates of each allele were analyzed by bidirectional DNA sequencing for length confirmation. Alleles were compared and numbered consecutively according to those identified within *C*. *parvum* isolates from domestic ruminants in Spain [25, 31–32]. Alleles were translated into numbers for multilocus analysis. Representative sequences generated in this study were deposited in the GenBank database under accession numbers KU729695 to KU729714.

The combination of alleles at each of the eleven VNTR loci and the GP60 gene defined the multilocus subtype (MLT) of each sample. Only isolates that were amplified at all loci were included in the multilocus analysis. The presence of two separated peaks for a specific locus differing by multiples of the repeat unit in CE electropherograms was designated a mixed infection, and the two potential MLTs were considered. Multilocus subtypes for isolates showing several alleles at more than one locus could not be determined, and these isolates were excluded from the genetic analyses. The discriminatory power of each individual marker and the multilocus approach for subtyping *C*. *parvum* isolates was assessed by calculating the Hunter-Gaston discriminatory index (HGDI), which estimates the probability of randomly picking two unrelated isolates and finding them to be different [33]. The VNTR diversity and confidence extractor software (V-DICE) available at the Health Protection Agency bioinformatics tools website was used for this purpose (http://www.hpa-bioinformatics.org.uk/cgi-bin/DICI/DICI.pl).

### Data analysis

Allelic linkage disequilibrium (LD) among different loci was assessed by measuring the standardized index of association (*I*_A_^S^) with the software LIAN v. 3.5 (http://guanine.evolbio.mpg.de/cgi-bin/lian/lian.cgi.pl), using the Monte Carlo method with 10,000 allele randomizations [34]. The index of association has a value of 0 for complete panmixia and a positive value if linkage disequilibrium is detected. The relationships among the MLTs were assessed using the eBURST algorithm (http://eburst.mlst.net/), which selects the most parsimonious patterns of subtype evolution and predicts founder(s) based on the allelic profile. The eBURST software v. 3 was used to identify clonal complexes, defined as clusters of closely related MLTs that were identical to each other on at least eleven loci (single-locus variants [SLVs]) [35]. The MLTs that were not related to any clonal complex were referred as singletons. In addition, STRUCTURE v.2.3.4 (http://pritchardlab.stanford.edu/structure.html) was used to identify distinct subpopulations on the basis of allelic frequencies and determine fractions of the MLT for each isolate that belongs to each subpopulation [36]. The most probable number of clusters was defined by calculating the *K* value as described elsewhere [37]. Four simulations runs were conducted for each *K* value using a length of burn-in of 10^4^ and 10^4^ replicates of Markov chain Monte Carlo simulation. GENETIX software (http://www.genetix.univ-montp2.fr/genetix/genetix.htm) was used to assess the robustness of the sub-structuring, calculating the Wright’s fixation index values (*F*st) for the different subpopulations [38]. The effective number of migrants between populations per generation (Nm) was estimated from *F*st values.

## Results

### Allele frequencies

Table 1 summarizes the numbers and sizes of alleles and HGDI values at each VNTR locus. A total of 101 *C*. *parvum* isolates from 46 farms were typable at all twelve loci and used for multilocus analysis. Loci exhibited various degrees of polymorphism, the finding of 7–9 alleles in the four most variable markers (ML2, Cgd6_5400, Cgd6_3940, and GP60) being remarkable. The latter were also the most discriminatory, with HGDI values ranging from 0.650 to 0.707. The percentage of specimens allocated to each allelic variant revealed that over 93% of isolates shared the same allele at CP47 and MSC6-7 loci, which explains the low discriminatory index for both markers. In contrast, alleles identified at the remaining loci were more evenly distributed within the *C*. *parvum* population, including two loci that only displayed three alleles (ML1, TP14). Five isolates showed a biallelic profile at 5B12, GP60, MSB, Cgd3_3850 or Cgd6_5400 loci, which evidenced the presence of intra-host mixed infections. Sequencing of isolates selected for fragment length confirmation revealed novel alleles at CP47 (420 pb), Cgd3_3850 (157 pb), Cgd1_3670 (235, 241, 259 and 277 pb), Cgd6_5400 (250, 262, 268, 271 and 289 pb) and Cgd6_3940 (324, 327, 333, 339 and 342 pb) loci. Additionally, alleles of 193 and 199 pb at the Cgd3_3850 locus differed by ten and four nucleotide polymorphisms outside the repeat region to the sequences deposited in Genbank under accession number KT806066 and KT806067, respectively. At the MSC6-7 locus, the allele with size of 516 pb differed by one nucleotide polymorphisms upstream the repeat region from the *C*. *parvum* reference sequence KM222523 from Genbank.

**Table 1:** Adjusted allele sizes and number allocation for each of eleven VNTR loci and the GP60 marker identified by automated capillary electrophoresis in *Cryptosporidium parvum* isolates from pre-weaned lambs.

Locus and adjusted fragment size (pb) (allele no.)^a^	No. of isolates (%) (n = 101)	No. of farms (n = 46)
**ML1 [HGDI = 0.531 (0.449–0.613)]** ^**b**^		
226 (1)	65 (64.3)	30
238 (2)	24 (23.8)	15
223 (3)	12 (11.9)	6
**TP14 [HGDI = 0.594 (0.530–0.658)]**		
324 (1)	56 (55.4)	27
333 (2)	26 (25.7)	16
342 (3)	19 (18.8)	13
**ML2 [HGDI = 0.702 (0.636–0.768)]**		
191 (2)	45 (44.5)	24
193 (9)	25 (24.7)	17
195 (15)	2 (1.9)	2
197 (10)	2 (1.9)	1
221 (12)	12 (11.9)	6
225 (16)	5 (4.9)	2
227 (3)	1 (0.9)	1
231 (5)	8 (7.9)	6
237 (8)	1 (0.9)	1
**5B12 [HGDI = 0.515 (0.433–0.597)]**		
167 (2)	27 (26.7)	16
169 (3)	65 (64.3)	33
171 (4)	8 (7.9)	6
165 (1) + 171	1 (0.9)	1
**MSB [HGDI = 0.605 (0.551–0.659)]**		
304 (1)	52 (51.5)	25
310 (5)	33 (32.7)	16
316 (2)	1 (0.9)	1
322 (3)	14 (13.9)	8
304 + 310	1 (0.9)	1
**CP47 [HGDI = 0.129 (0.043–0.215)]**		
417 (1)	94 (93.1)	43
420 (3)	2 (1.9)	1
423 (4)	5 (4.9)	4
**MSC6-7 [HGDI = 0.038 (0.000–0.089)]**		
516 (3)	99 (98)	45
549 (1)	2 (1.9)	1
**Cgd3_3850 [HGDI = 0.591 (0.510–0.673)]**		
157 (7)	24 (23.8)	10
163 (2)	58 (57.4)	29
181 (3)	3 (2.9)	2
193 (4)	10 (9.9)	5
199 (5)	5 (4.9)	4
157 + 193	1 (0.9)	1
**Cgd1_3670 [HGDI = 0.444 (0.335–0.552)]**		
235 (5)	73 (72.3)	33
241 (6)	5 (4.9)	3
247 (7)	1 (0.9)	1
259 (8)	1 (0.9)	1
265 (2)	15 (14.8)	8
277 (9)	6 (5.9)	3
**Cgd6_5400 [HGDI = 0.707 (0.643–0.771)]**		
250 (4)	10 (9.9)	6
262 (5)	5 (4.9)	5
268 (6)	3 (2.9)	3
271 (7)	48 (47.5)	21
277 (1)	7 (6.9)	5
283 (2)	24 (23.8)	14
289 (8)	3 (2.9)	2
277 + 283	1 (0.9)	1
**Cgd6_3940 [HGDI = 0.692 (0.621–0.762)]**		
312 (1)	24 (23.8)	13
324 (4)	4 (3.9)	3
327 (5)	3 (2.9)	2
330 (2)	1 (0.9)	1
333 (6)	8 (7.9)	5
336 (3)	8 (7.9)	5
339 (7)	51 (50.5)	27
342 (8)	2 (1.9)	2
**GP60 [HGDI = 0.650 (0.572–0.729)]**		
339 (7)	54 (53.5)	29
342 (8)	8 (7.9)	5
345 (2)	23 (22.8)	16
348 (9)	3 (2.9)	2
351 (3)	8 (7.9)	7
354 (4)	2 (1.9)	2
360 (10)	1 (0.9)	1
339 + 345	1 (0.9)	1
339 + 351	1 (0.9)	1

^a^ Alleles were compared and numbered consecutively according to those identified within *Cryptosporidium* isolates from calves and lambs by Quílez et al. [25, 31] and Ramo et al. [32]

^b^ Hunter-Gaston discriminatory power [discriminatory index (95% confidence interval)]

Comparison with a previous study in calves in northern Spain using the same 12-loci typing approach showed differences in the distribution of alleles at most loci [32]. Comparative charts are provided as supplementary material (S1 and S2 Figs). Namely, differences were seen in the identity of major alleles at ML1 (226 bp *versus* 238 bp), ML2 (191 and 193 bp *vs* 231 and 233 bp), MSB (304 and 310 bp *vs* 322 bp), MSC6-7 (516 bp *vs* 549 bp), Cgd3_3850 (157 and 163 bp *vs* 193 bp), Cgd1_3670 (235 bp *vs* 265 bp), Cgd6_5400 (271 and 283 bp *vs* 277 bp), Cgd6_3940 (312 and 339 bp *vs* 336 bp), and GP60 (339 and 345 bp *vs* 351 bp) loci for isolates from lambs and calves, respectively. Additionally, most loci were much more discriminatory for typing isolates from lambs. In particularly, some markers reported as monomorphic (MSB) or hardly informative in calves (ML1, Cgd6_3940) provided a relatively high HGDI value in lambs.

### Multilocus subtypes

The composition and frequency of multilocus subtypes (MLTs) identified among *C*. *parvum* isolates from lambs is listed in Table 2. A complete twelve-locus subtype was obtained from 100 specimens and 74 MLTs were identified. Four isolates from different farms showed a biallelic profile at one locus and were scored each as having two different MLTs. One additional isolate showed evidence of mixed infection at two loci and was removed from the multilocus analysis. Most MLTs (64/74) were unique to individual farms, while each of nine MLTs were concurrently detected on two farms and a single MLT was simultaneously identified on five fams. Nevertheless, most isolates originating at the same farm were not identical, as demonstrated by the presence of multiple MLTs in 28/46 farms, being relevant the finding of two farms each harbouring four MLTs and a single farm where seven MLTs were identified. Comparison with haplotypes previously reported in Spanish cattle farms showed that only one MLT was shared by lambs (MLT-2) and calves (MLT-27) [32]. The S1 Table provides details on the allelic profile of each isolate used in this study and includes data related to its origin. The HGDI value of the twelve-satellite typing method was 0.988 (95% CI, 0.979−0.996)]. The analysis of the number of MLTs and HGDI value generated by different combinations of markers is showed in the S2 Table. The combination of only the two most informative loci (ML2, Cgd6_5400) notably improved the discriminatory power of each individual marker and identified up to 22 MLTs. The resolution was also significantly increased by the combination of the three and four most informative markers, which provided 17 and 12 additional MLTs, respectively, although up to ten markers were needed to identify all MLTs.

**Table 2:** Multilocus subtypes (MLTs) of *Cryptosporidium parvum* isolates from pre-weaned lambs based on the combination of alleles at eleven VNTR loci and the GP60 marker.

MLT	Allele at locus^a^												No. of isolates (n = 100)^b^	No. of farms (n = 46)
	MSC6-7	CP47	Cgd1_ 3670	5B12	ML1	Cgd3_3850	TP14	MSB	Cgd6_ 3940	Cgd6_ 5400	ML2	GP60		
**1**	1	1	2	3	1	4	1	3	1	1	9	10	1	1
**2**	1	1	2	3	2	4	1	3	3	1	5	3	1	1
**3**	3	1	2	2	1	5	3	5	8	5	2	9	1	1
**4**	3	1	2	3	1	2	1	1	7	7	2	2	3	1
**5**	3	1	2	3	1	2	1	5	7	1	9	7	1	1
**6**	3	1	2	3	1	2	1	5	7	2	9	7	1	1
**7**	3	1	2	3	1	2	2	5	7	7	2	2	1	1
**8**	3	1	2	3	1	2	2	5	7	7	2	7	1	1
**9**	3	1	2	3	2	2	1	1	7	7	2	2	2	2
**10**	3	1	2	3	2	2	1	1	7	7	2	3	1	1
**11**	3	1	5	1	3	7	1	5	1	7	2	9	1	1
**12**	3	1	5	2	1	2	1	5	7	7	2	7	3	1
**13**	3	1	5	2	1	2	2	3	7	7	12	7	1	1
**14**	3	1	5	2	1	7	1	1	1	7	9	3	1	1
**15**	3	1	5	2	1	7	1	1	7	4	2	7	1	1
**16**	3	1	5	2	2	2	1	1	1	7	5	7	2	2
**17**	3	1	5	2	2	2	1	1	1	7	9	7	2	1
**18**	3	1	5	2	2	2	1	1	7	5	9	7	1	1
**19**	3	1	5	2	2	2	2	1	1	7	5	7	1	1
**20**	3	1	5	2	2	7	2	5	1	8	9	7	3	2
**21**	3	1	5	2	3	2	1	5	7	2	2	7	1	1
**22**	3	1	5	2	3	7	1	3	1	7	2	9	1	1
**23**	3	1	5	3	1	2	1	1	6	1	2	3	1	1
**24**	3	1	5	3	1	2	1	1	6	2	2	2	3	2
**25**	3	1	5	3	1	2	1	1	6	2	2	3	1	1
**26**	3	1	5	3	1	2	1	1	7	7	9	7	4	2
**27**	3	1	5	3	1	2	2	1	2	2	2	2	1	1
**28**	3	1	5	3	1	2	2	1	6	2	2	2	2	2
**29**	3	1	5	3	1	2	2	1	7	7	2	2	1	1
**30**	3	1	5	3	1	2	2	1	7	7	15	2	1	1
**31**	3	1	5	3	1	2	2	1	8	2	2	2	1	1
**32**	3	1	5	3	1	2	2	1	8	2	2	7	1	1
**33**	3	1	5	3	1	2	2	3	7	6	2	7	1	1
**34**	3	1	5	3	1	2	2	3	7	7	12	7	9	5
**35**	3	1	5	3	1	4	2	5	7	4	9	7	1	1
**36**	3	1	5	3	1	4	3	5	1	4	9	7	1	1
**37**	3	1	5	3	1	4	3	5	7	1	9	7	1	1
**38**	3	1	5	3	1	4	3	5	7	2	9	7	1	1
**39**	3	1	5	3	1	4	3	5	7	4	2	7	1	1
**40**	3	1	5	3	1	4	3	5	7	4	9	2	1	1
**41**	3	1	5	3	1	7	1	1	1	7	3	7	1	1
**42**	3	1	5	3	1	7	1	1	1	7	15	2	1	1
**43**	3	1	5	3	1	7	1	1	1	7	16	7	3	2
**44**	3	1	5	3	1	7	1	1	6	7	16	7	1	1
**45**	3	1	5	3	1	7	1	1	7	7	16	7	1	1
**46**	3	1	5	3	1	7	2	1	6	2	2	2	1	1
**47**	3	1	5	3	1	7	3	1	1	7	2	7	1	1
**48**	3	1	5	3	2	2	1	1	7	7	2	3	1	1
**49**	3	1	5	3	2	4	3	1	4	2	10	8	2	1
**50**	3	1	5	3	3	2	1	5	7	2	2	3	1	1
**51**	3	1	5	3	3	2	1	5	7	2	2	7	2	1
**52**	3	1	5	3	3	2	1	5	7	2	5	7	2	2
**53**	3	1	5	3	3	2	1	5	7	2	9	7	1	1
**54**	3	1	5	3	3	7	3	5	1	1	2	4	1	1
**55**	3	1	5	4	1	2	1	1	7	2	2	3	1	1
**56**	3	1	5	4	1	2	1	1	7	2	2	7	1	1
**57**	3	1	5	4	1	5	3	1	5	2	9	3	1	1
**58**	3	1	5	4	1	5	3	1	5	2	12	8	2	1
**59**	3	1	5	4	3	7	1	5	1	7	2	9	1	1
**60**	3	1	6	2	3	3	1	1	4	4	2	2	1	1
**61**	3	1	7	4	1	5	1	2	3	4	8	8	1	1
**62**	3	1	8	3	1	2	1	1	7	5	9	7	1	1
**63**	3	1	9	2	2	7	1	5	3	5	2	8	2	2
**64**	3	1	9	2	2	7	1	5	3	6	2	4	1	1
**65**	3	1	9	2	2	7	1	5	3	6	2	8	1	1
**66**	3	1	9	3	2	2	1	5	7	1	9	7	1	1
**67**	3	1	9	4	2	2	1	5	7	2	9	7	1	1
**68**	3	3	2	2	1	7	3	1	3	7	9	7	1	1
**69**	3	3	2	2	2	7	3	1	3	7	9	7	1	1
**70**	3	4	5	3	3	7	3	5	1	1	2	2	1	1
**71**	3	4	6	2	1	3	3	5	1	7	2	2	1	1
**72**	3	4	6	2	2	2	3	1	4	4	2	2	1	1
**73**	3	4	6	3	1	2	2	1	1	4	2	3	1	1
**74**	3	4	6	4	3	3	3	1	1	4	2	2	1	1

^a^ The number allocation for alleles is indicated in Table 1

^b^ Only those isolates typable at all loci were used for multilocus analyses. Samples with mixed infections at a single locus were allocated to the corresponding MLT

### Population analysis

**Fig 2:**
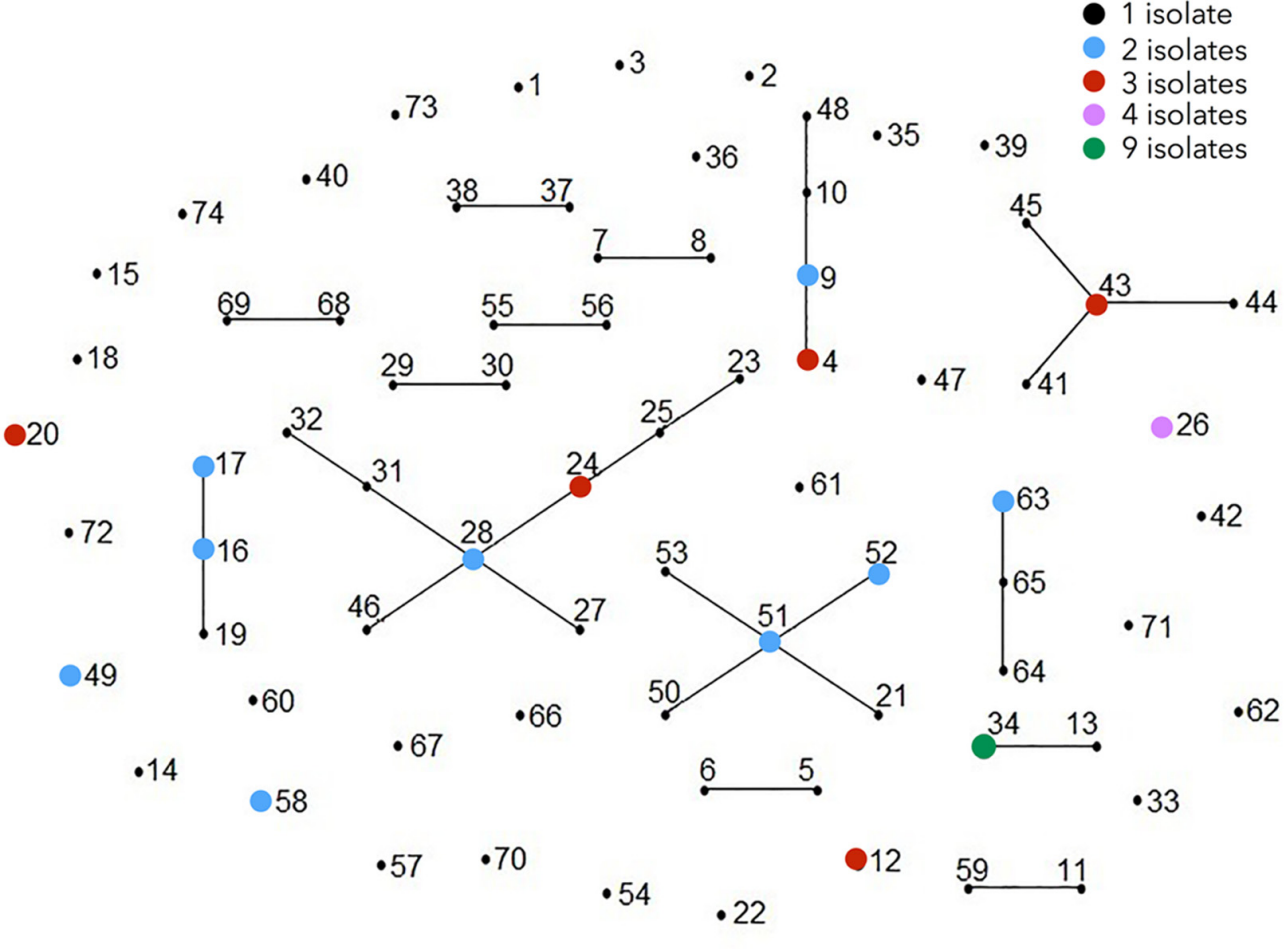
Single-locus variant eBURST network for 74 multilocus subtypes (MLTs) identified among 100 *Cryptosporidium parvum* isolates from lambs. Each MLT is represented by a dot, which is colored according to the number of isolates as shown in the key. Single-locus variants are joined by lines. Distance between dots is random and does not provide additional information. The allelic profile of each MLT is indicated in Table 2.

Linkage disequilibrium tests performed on the whole sample were highly significant (*I*_A_^S^: 0.0703; *P*<10^−4^) and the pairwise variance (V_D_: 4.3845) was greater than the 95% critical value (*L*: 2.6948). Values remained positive when counting only once all repeated MLTs (*I*_A_^S^: 0.0512; *P*<10^−4^), which shows that linkage disequilibrium is due to clonal evolution rather than to epidemic expansion of a few particular haplotypes. The eBURST analysis placed 43 MLTs into 14 clonal complexes, along with 31 MLTs from 21 farms with singleton status. Eight clonal complexes linked only 2 MLTs, and the remaining linked less than 9 MLTs (Fig 2). The evolutionary relationships among specimens were also investigated using a Bayesian clustering method called STRUCTURE. The most probable value for the number of clusters (*K*) according to Evanno et al. [37] was calculated using the software Structure Harvester v0.6.93 in the range of *K* = 1 to *K* = 46 (http://taylor0.biology.ucla.edu/structureHarvester/) [39]. The maximum delta K (ΔK) value was obtained for *K* = 26 (12760.15), but high values were also found for *K* = 29 (7816.89), *K* = 36 (7364.00) and *K* = 39 (5511.17), which suggest a weak genetic clustering for the isolates obtained in the 46 farms (S3 Table, S3 Fig). These results were supported by *F* statistics, with a global *F*st value of 0.67 (95% confidence interval range 0.59438 to 0.73904 after 1000 bootstraps), indicating limited interactions among specimens in the different farms. The pairwise *F*st analysis showed that 153/1,035 combinations reached the maximum possible value (*F*st = 1), while only 20 pairs of farms provided *F*st = 0. The estimation of the number of migrants per generation among farms (Nm) provided very low values, as expected for non-genetically related groups, with Nm ≥ 1 for only 14/1,015 combinations.

## Discussion

The 12-loci typing approach used in this study unraveled a high genetic diversity of *C*. *parvum* in sheep farms at a small geographical scale. The technique identified 74 MLTs within 100 isolates, as compared to 48 MLTs within 122 isolates previously seen in lambs using a 6-loci typing scheme in a more extensive area in Spain [25]. The typing tool also provided a remarkable typeability, with 101/113 specimens amplifying at all twelve loci. The analysis of allelic variability at individual loci showed that most of them exhibited a relatively high HGDI value and thus contributed in some degree to the discriminatory power of the technique. In fact, both the discriminatory index and resolution of individual markers were notably improved by the multilocus approach, with an HGDI value over 0.9 being achieved with the combination of only two markers and nearly 70% of the total MLTs being identified by combining four loci. Only CP47 and MSC6-7 could be excluded with no reduction in the numbers of MLTs, which is in agreement with previous observations designating both markers among the less polymorphic for *C*. *parvum* from humans and cattle [32, 40, 41]. It is worth noting that two novel loci not previously used for typing specimens from lambs (Cgd6_5400, Cgd6_3940) were among the most informative and thus should be considered for subsequent multilocus analyses.

Results of this study revealed the distinctiveness of *C*. *parvum* isolates infecting lambs in this geographical area. Comparison with previous observations in dairy cattle using the same subtyping scheme showed differences in the identity of major alleles at most loci, and a single MLT was shared between isolates from lambs and calves [32]. Differences also applied to the HGDI value, with most loci being much more discriminatory for typing ovine specimens, which could be explained by a more even distribution of the different allelic variants among the parasite population. Previous studies with different sets of molecular markers have also supported the uniqueness of *C*. *parvum* in sheep in northeastern Spain. Preliminary research by GP60 sequencing showed a remarkable predominance of subtypes belonging to the family IId in lambs, whereas calves are preferentially infected by IIa subtypes [22, 42]. Subsequent studies by fragment analysis revealed that differences also applied to other VNTR markers, with the presence of host-associated subpopulations for *C*. *parvum* infecting lambs and calves [25, 31]. The singularity of ovine specimens was also seen in Italy, where not a single MLT was shared between sheep and goats and only 3 MLTs from sheep were also found in calves [17]. In contrast, no evidence of host association was reported in Scotland, where most MLTs identified within 11 ovine isolates had previously been found in cattle or humans [14]. Nevertheless, observations in other Spanish areas where livestock share grazing grounds and facilities have shown that calves, lambs and goat kids are mostly infected with subtypes in the IIa family, which demonstrates the relevance of management factors to explain differences in the distribution of *C*. *parvum* subtypes [23, 24].

Another relevant finding in this study was the uniqueness of most MLTs to individual farms (64/74), which strongly indicates that cryptosporidial infection is mainly transmitted within sheep farms through the expansion of genetically unique strains in lambing areas, with the environmentally-resistant oocysts maintaining infections between lambing periods and herd-to-herd transmission playing a secondary role. This observation is consistent with the endemicity of cryptosporidiosis reported in ovine flocks in this province, where high infections rates were found and overcrowding and poor hygienic conditions of the lambing facilities have been reported as risk factors [26]. This finding could also be supported by the predominance of a rearing system where most producers generate their own replacement females by retaining ewe lambs, while introduction of new animals in herds is an uncommon event.

The presence of multiple MLTs on more than a half of farms evidenced a significant intra-farm genetic diversity, the finding of up to seven MLTs in a farm being remarkable. In contrast, only five isolates showed a biallelic profile at one or more loci, which indicates a limited intra-host variability, much lower than that previously reported in livestock in northeastern Spain, where more than 25% of sheep (19/73) and cattle farms (22/61) had animals concurrently infected by genetically distinct *C*. *parvum* strains [25, 31]. In cattle, the occurrence of mixed infections has been related to frequent animal movement between farms [16, 43, 44]. However, the opportunities of transmission between sheep herds in the current study were partly limited by the above-mentioned farming system, which suggests that additional factors such as frequent mutations or genetic exchange through recombination could explain the extensive intra-herd heterogeneity. Studies in other countries have reported a low rate of mixed infections within cattle farms. Most isolates originating at the same farm were identical in the United States and the United Kingdom, and the majority of calves (109/118) were shedding single alleles at each locus in the latter country [40, 45]. Similarly, only 11/277 specimens from calves had mixed MLTs in Ireland, a finding that the authors related to the geographical isolation of the island [44].

The distinctiveness of MLTs was also consistent with results of evolutionary descent by the algorithm eBURST which indicated a high degree of genetic divergence, with over 41% MLTs appearing as singletons along with a high number of clonal complexes, each linking a few MLTs. It is significant to note that most clonal complexes linking only two MLTs (7/8) were unique to individual farms, which indicates that SLV are common among isolates circulating in sheep farms. The results of STRUCTURE analysis and *F* statistics were also helpful in revealing the genetic remoteness of *C*. *parvum* isolates, which were not easily classified by the Bayesian clustering method. In fact, analyses of combined data indicated that *K* = 26 ancestral types best explained the current population structure, but similar profiles were obtained using other high *K* values and no ancestral population size was chosen.

Most studies on the population structure of *C*. *parvum* have been conducted in humans or cattle, but data with specimens from sheep are extremely limited. A panmictic structure typical of populations where genetic exchange occurs at random with limited or no sub-structuring has been reported in cattle farms in Ireland [44]. In contrast, a prevalent pattern of clonality was found in Italy with specimens from humans and livestock, including a reduced number of ovine samples [17]. Other studies have reported a more flexible reproductive strategy with the co-occurrence of panmictic, clonal and epidemic structure [2]. Evidence of these variations has been found in the United States, where *C*. *parvum* is essentially panmictic in the Midwest, but epidemic in Minessota [40]. Similarly, *C*. *parvum* is panmictic in cattle but epidemic in humans in Scotland [14], or panmictic in Dumfriesshire and Aberdeenshire, but epidemic in Orkney and Thurso [16]. Variations in the prevailing pathways according to geographic and host factors have also been found in Spain, where *C*. *parvum* population was predominantly panmictic in cattle farms in the north, but epidemic in sheep and goat farms in a more extensive area in the northeast of the country [25, 32].

In the current study, linkage analyses showed that alleles in the population are in linkage disequilibrium. However, this condition was maintained when isolates exhibiting the same MLT were scored as a single individual, excluding that LD could arise from the clonal expansion of one or more MLT to produce epidemic clones, and revealing an overall clonal structure. This prevalent pattern of clonality could be explained by the lack of outcrossing opportunities due to spatial isolation imposed by husbandry practices, although the high level of intra-herd variability and the low value of the index of association suggest that genetic exchange is also occurring at some significant level within some farms. A similar view was advanced by Drumo et al. [17], who reported genetic exchange within an overall clonal population of *C*. *parvum* in livestock in Italy.

In summary, the data presented in this study have provided evidence of the genetic richness and distinct identity of *C*. *parvum* strains circulating in sheep farms in a confined area. The VNTR typing approach is a suitable tool for epidemiological tracking, although a number of factors should be taken into consideration for attributing acquired *Cryptosporidium* infections to specific sources, including local factors, management systems and host information. Our results also indicate a predominantly clonal structure of *C*. *parvum* in the sheep population in this discrete region, although comparison with previous research demonstrates a complex epidemiology with the occurrence of different reproduction patterns in livestock farms in Spain, which supports the need of further investigations with more exhaustive sampling within farms.

## Supporting Information

S1 Fig Frequency distribution of alleles at different microsatellite loci identified by fragment typing in *Cryptosporidium parvum* isolates from 101 lambs in this study and 104 calves in a previous study in northern Spain [32]. (TIFF)

S2 Fig Frequency distribution of alleles at different minisatellite loci identified by fragment typing in *Cryptosporidium parvum* isolates from 101 lambs in this study and 104 calves in a previous study in northern Spain [32]. (TIFF)

S3 Fig Graph of the delta *K* (ΔK) values to estimate the number of ancestral populations in *Cryptosporidium parvum* isolates from lambs using the Evanno method implemented in Structure Harvester [39]. Data were estimated using eleven mini- and microsatellite loci and the GP60 marker. ΔK = mjL′′(K)j/s[L(K)], where m = mean of the absolute values of L′′(K), s = SD of L(K).
(TIFF)

S1 Table Allelic profile for 101 *C*. *parvum* isolates used in this study. Samples exhibiting a mixed infection at a single locus are duplicated and allocated to the corresponding multilocus subtype (MLT). One sample showing a mixed infection at two loci was excluded from the genetic analysis.
(XLSX)

S2 Table Number of multilocus types (MLTs) and HGDI value (discriminatory index, 95% confidence interval) generated by the combination of different mini- and microsatellite markers. Combinations were determined by the sequential addition of markers with decreasing individual HGDI value from left to right. The inclusion of two additional markers (CP47, MSC6-7) did not increase the number of MLTs.
(DOCX)

S3 Table Estimation of the number of ancestral populations (*K*) in *Cryptosporidium parvum* isolates from lambs using the Evanno method implemented in Structure Harvester [39]. Table output of the Evanno method results. Green highlight is performed dynamically on the website and shows the largest value in the Delta K column.
(DOCX)
