# Supplementary figures and images for: Intra-Species Genetic Diversity and Clonal Structure of Cryptosporidium parvum in Sheep Farms in a Confined Geographical Area in Northeastern Spain
Source: PLoS One. 2016 May 13;11(5):e0155336. doi: 10.1371/journal.pone.0155336 (PMC4866762; doi:10.1371/journal.pone.0155336)

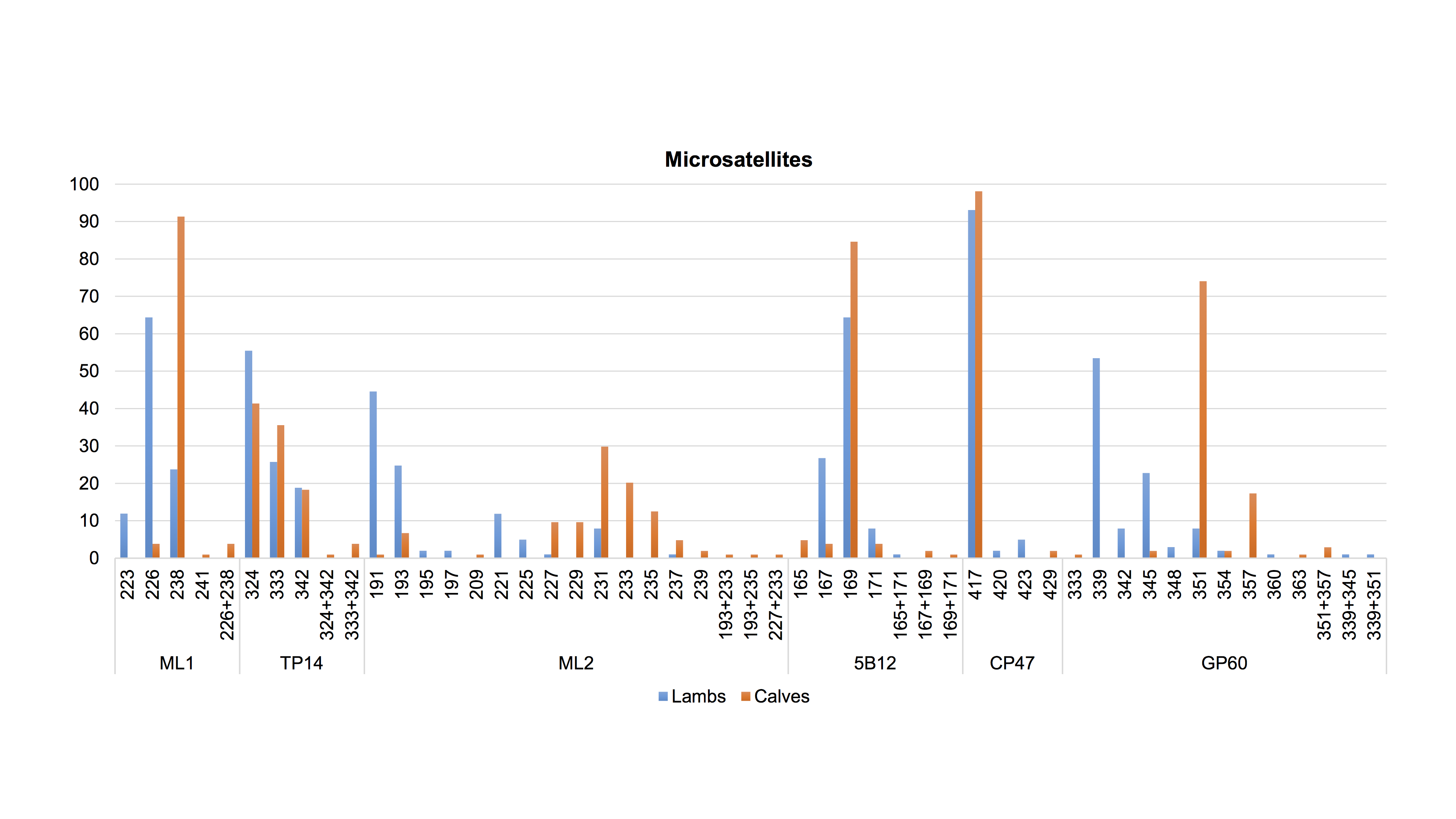

Supplement: S1 Fig — (TIFF) [file pone.0155336.s001.tiff]

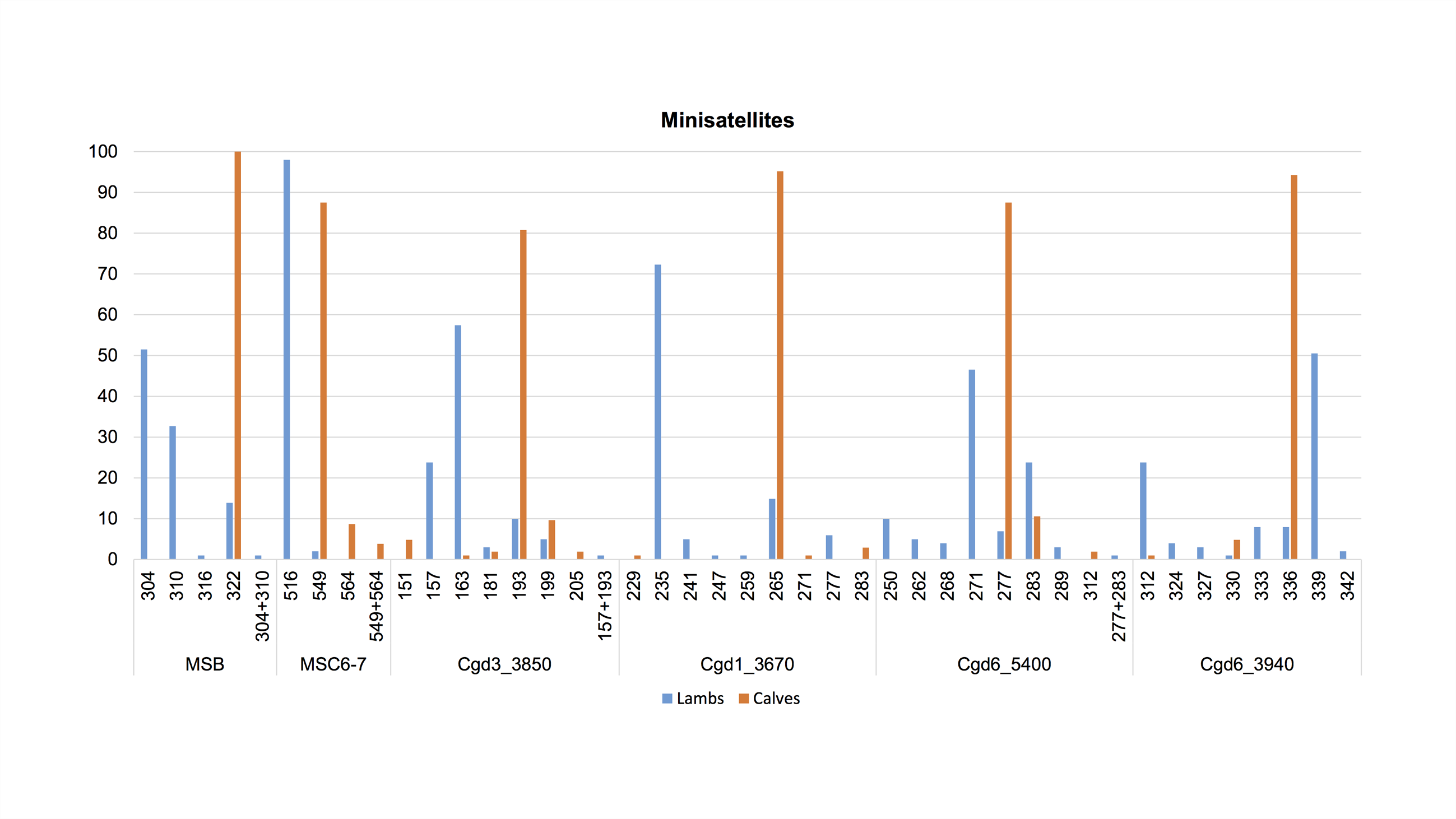

Supplement: S2 Fig — (TIFF) [file pone.0155336.s002.tiff]

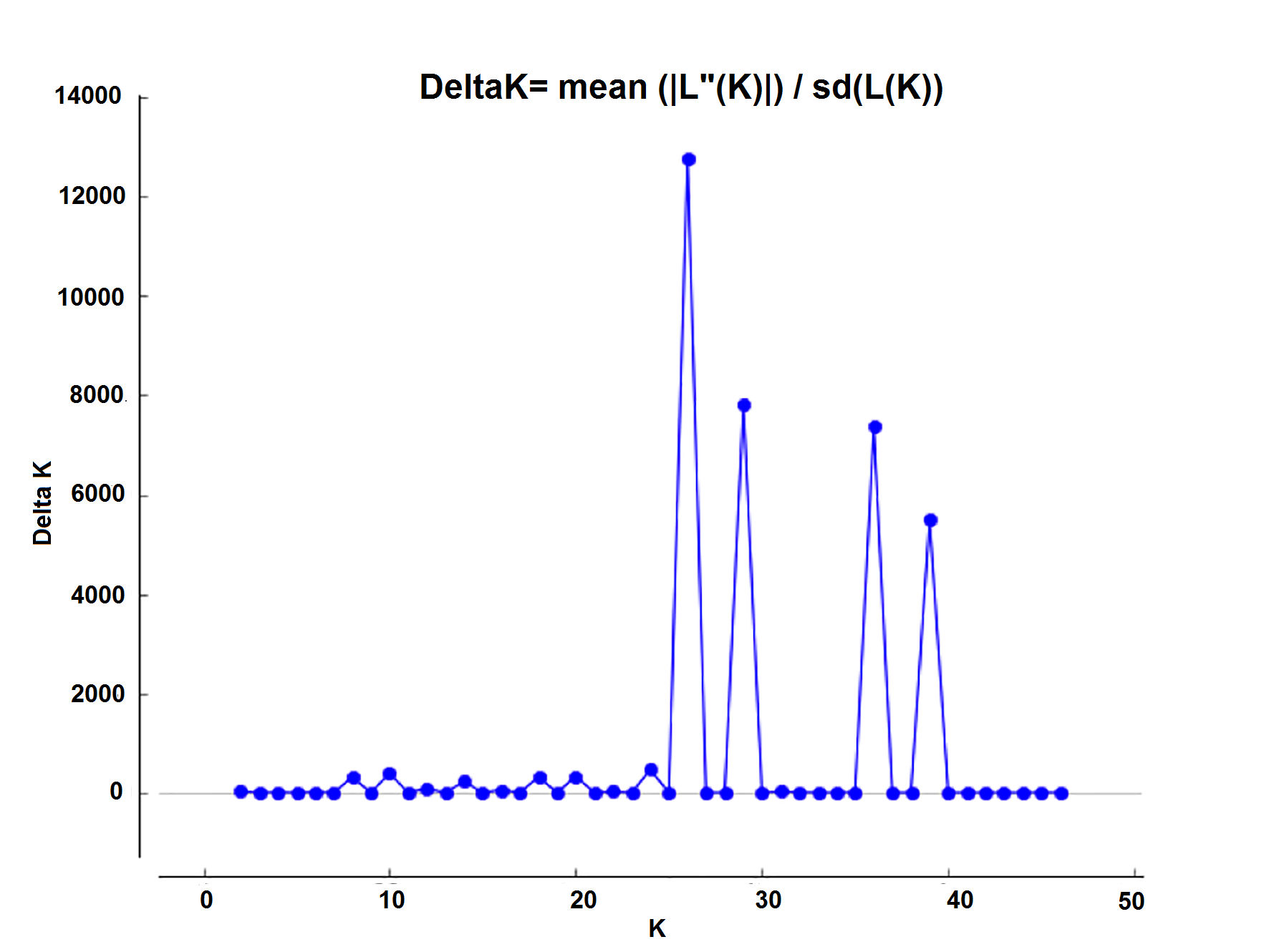

Supplement: S3 Fig — Data were estimated using eleven mini- and microsatellite loci and the GP60 marker. ΔK = mjL′′(K)j/s[L(K)], where m = mean of the absolute values of L′′(K), s = SD of L(K). (TIFF) [file pone.0155336.s003.tiff]
