# Supplementary material for: Intra-Species Genetic Diversity and Clonal Structure of Cryptosporidium parvum in Sheep Farms in a Confined Geographical Area in Northeastern Spain
Source: PLoS One. 2016 May 13;11(5):e0155336. doi: 10.1371/journal.pone.0155336 (PMC4866762; doi:10.1371/journal.pone.0155336)
Supplement: S2 Table — Combinations were determined by the sequential addition of markers with decreasing individual HGDI value from left to right. The inclusion of two additional markers (CP47, MSC6-7) did not increase the number of MLTs. (DOCX) [file pone.0155336.s005.docx]

| **Cgd6_5400** | **ML2** | **Cgd6_3940** | **GP60** | **MSB** | **TP14** | **Cgd3_3850** | **ML1** | **5B12** | **Cgd1_3670** |
| --- | --- | --- | --- | --- | --- | --- | --- | --- | --- |
| 22 MLTs (0.922; 95% CI, 0.899-0.945) | |  |  |  |  |  |  |  |  |
| 39 MLTs (0.961; 95%CI, 0.946-0.976) | | |  |  |  |  |  |  |  |
| 51 MLTs (0.976; 95% CI, 0.965-0.986) | | | |  |  |  |  |  |  |
| 56 MLTs (0.978; 95% CI, 0.968-0.988) | | | | |  |  |  |  |  |
| 63 MLTs (0.983; 95% CI, 0.973-0.992) | | | | | |  |  |  |  |
| 64 MLTs (0.983; 95% CI, 0.973-0.993) | | | | | | |  |  |  |
| 70 MLTs (0.985; 95% CI, 0.976-0.995) | | | | | | | |  |  |
| 73 MLTs (0.987; 95% CI, 0.979-0.996) | | | | | | | | |  |
| 74 MLTs (0.988; 95% CI, 0.979-0.996) | | | | | | | | | |
