# Supplementary material for: Intra-Species Genetic Diversity and Clonal Structure of Cryptosporidium parvum in Sheep Farms in a Confined Geographical Area in Northeastern Spain
Source: PLoS One. 2016 May 13;11(5):e0155336. doi: 10.1371/journal.pone.0155336 (PMC4866762; doi:10.1371/journal.pone.0155336)
Supplement: S3 Table — Table output of the Evanno method results. Green highlight is performed dynamically on the website and shows the largest value in the Delta K column. (DOCX) [file pone.0155336.s006.docx]

| **K** | **Reps** | **Mean LnP(K)** | **Stdev LnP(K)** | **Ln'(K)** | **\|Ln''(K)\|** | **Delta K** |
| --- | --- | --- | --- | --- | --- | --- |
| 1 | 3 | -1284.900000 | 0.173205 | — | — | — |
| 2 | 3 | -1164.333333 | 1.703917 | 120.566667 | 52.500000 | 30.811359 |
| 3 | 3 | -1096.266667 | 2.311565 | 68.066667 | 0.600000 | 0.259564 |
| 4 | 3 | -1027.600000 | 9.913627 | 68.666667 | 97.200000 | 9.804686 |
| 5 | 3 | -1056.133333 | 185.475722 | -28.533333 | 154.500000 | 0.832993 |
| 6 | 3 | -930.166667 | 6.459360 | 125.966667 | 88.500000 | 13.701047 |
| 7 | 3 | -892.700000 | 14.918780 | 37.466667 | 18.633333 | 1.248985 |
| 8 | 3 | -873.866667 | 15.337644 | 18.833333 | 4774.400000 | 311.286394 |
| 9 | 3 | -5629.433333 | 8297.447505 | -4755.566667 | 9585.933333 | 1.155287 |
| 10 | 3 | -799.066667 | 12.499733 | 4830.366667 | 4820.800000 | 385.672228 |
| 11 | 3 | -789.500000 | 39.237355 | 9.566667 | 70.466667 | 1.795908 |
| 12 | 3 | -850.400000 | 134.010186 | -60.900000 | 11269.700000 | 84.095846 |
| 13 | 3 | -12181.00000 | 19690.827925 | -11330.60000 | 22680.800000 | 1.151846 |
| 14 | 3 | -830.800000 | 52.020477 | 11350.200000 | 11642.533333 | 223.806740 |
| 15 | 3 | -1123.133333 | 250.406816 | -292.333333 | 370.866667 | 1.481057 |
| 16 | 3 | -1044.600000 | 208.712793 | 78.533333 | 12401.800000 | 59.420411 |
| 17 | 3 | -13367.86666 | 11303.983180 | -12323.26666 | 24589.633333 | 2.175307 |
| 18 | 3 | -1101.500000 | 95.489109 | 12266.366667 | 30115.066667 | 315.376977 |
| 19 | 3 | -18950.20000 | 30746.508290 | -17848.70000 | 35704.500000 | 1.161254 |
| 20 | 3 | -1094.400000 | 58.867393 | 17855.800000 | 19374.700000 | 329.124476 |
| 21 | 3 | -2613.300000 | 2584.890448 | -1518.900000 | 2584.600000 | 0.999888 |
| 22 | 3 | -1547.600000 | 594.028257 | 1065.700000 | 12213.766667 | 20.560919 |
| 23 | 3 | -12695.66666 | 19763.25134 | -11148.06666 | 22599.533333 | 1.143513 |
| 24 | 3 | -1244.200000 | 70.842643 | 11451.466667 | 32650.933333 | 460.893780 |
| 25 | 3 | -22443.66666 | 18491.386249 | -21199.46666 | 42357.633333 | 2.290668 |
| 26 | 3 | -1285.500000 | 3.386739 | 21158.166667 | 43215.300000 | 12760.151280 |
| 27 | 3 | -23342.63333 | 20781.107605 | -22057.13333 | 39678.166667 | 1.909338 |
| 28 | 3 | -5721.600000 | 7684.330187 | 17621.033333 | 13184.666667 | 1.715786 |
| 29 | 3 | -1285.233333 | 0.709460 | 4436.366667 | 5545.773333 | 7816.894829 |
| 30 | 5 | -2394.640000 | 2512.361844 | -1109.406667 | 2240.921667 | 0.891958 |
| 31 | 4 | -1263.125000 | 51.003096 | 1131.515000 | 1155.340000 | 22.652350 |
| 32 | 4 | -1286.950000 | 3.445287 | -23.825000 | 24.375000 | 7.074883 |
| 33 | 4 | -1286.400000 | 2.415229 | 0.550000 | 9.350000 | 3.871268 |
| 34 | 4 | -1276.500000 | 15.854547 | 9.900000 | 19.850000 | 1.252007 |
| 35 | 4 | -1286.450000 | 1.250333 | -9.950000 | 11.825000 | 9.457478 |
| 36 | 4 | -1284.575000 | 0.974252 | 1.875000 | 7174.400000 | 7364.009627 |
| 37 | 4 | -8457.100000 | 11875.305659 | -7172.525000 | 2282.900000 | 0.192239 |
| 38 | 4 | -17912.52500 | 19392.248471 | -9455.425000 | 26083.525000 | 1.345049 |
| 39 | 4 | -1284.425000 | 3.017035 | 16628.100000 | 16627.408333 | 5511.175213 |
| 40 | 3 | -1283.733333 | 1.320353 | 0.691667 | 2.125000 | 1.609417 |
| 41 | 3 | -1285.166667 | 1.514376 | -1.433333 | 2.533333 | 1.672857 |
| 42 | 3 | -1284.066667 | 1.497776 | 1.100000 | 2.166667 | 1.446589 |
| 43 | 3 | -1285.133333 | 1.159023 | -1.066667 | 1.833333 | 1.581793 |
| 44 | 3 | -1284.366667 | 1.357694 | 0.766667 | 2.033333 | 1.497637 |
| 45 | 3 | -1285.633333 | 0.702377 | -1.266667 | 3.133333 | 4.461043 |
| 46 | 3 | -1283.766667 | 2.267892 | 1.866667 | 4.833333 | 2.131201 |
| 47 | 3 | -1286.733333 | 4.200397 | -2.966667 | — | — |
